# Innovative modeling: a diet-induced quail model for progressive pathological changes in uric acid metabolism disorders

**DOI:** 10.3389/fnut.2025.1612479

**Published:** 2025-07-23

**Authors:** Yi Xu, Yu Wang, Fujun Gao, Chengjin Lu, Shujia Liu, Siying Chen, Xiaomeng Zhang, Zhijian Lin, Bing Zhang

**Affiliations:** School of Chinese Materia Medica, Beijing University of Chinese Medicine, Beijing, China

**Keywords:** diet-induced, hyperuricemia, uric acid nephropathy, gouty arthritis, quail models

## Abstract

**Background:**

Diseases associated with uric acid metabolism disorders, primarily hyperuricemia, uric acid nephropathy, and gouty arthritis, are increasingly prevalent worldwide. Recent research suggests that hyperuricemia, uric acid nephropathy, and gouty arthritis can be regarded as distinct phases of the same disease, characterized by elevated serum uric acid levels and the progressive pathological manifestations observed in clinical settings. Animal models play a crucial role in investigating disease mechanisms and therapeutic interventions. However, there are currently few animal models available that can accurately simulate human uric acid metabolism disorders on the same animal, particularly those exhibiting progressive pathological features.

**Methods:**

This study established a quail model of urate metabolism disorder using 25-day-old male Defaike quails via dietary induction. The diet consisted of high-calcium/high-purine components, including 20% yeast extract and 30% bone extract powder, supplemented with 15 mL of 10% fructose water daily for 30 days. The model effectively recapitulated three progressive pathological stages: (1) Hyperuricemia; (2) Hyperuricemia with urate nephropathy; and (3) Hyperuricemia with gouty arthritis. In the simple hyperuricemia stage, serum uric acid levels significantly increased after 10 days of intervention, with no significant deposition of monosodium urate (MSU) crystals observed in the kidneys or synovial fluid. In the second stage, hyperuricemia combined with uric acid nephropathy, renal MSU crystals were deposited after 20 days, while serum uric acid levels remained elevated, and serum creatinine (CRE) and blood urea nitrogen (BUN) significantly increased, accompanied pathological changes in renal tissue. In the final stage, hyperuricemia combined with gouty arthritis, MSU crystals were deposited in joint synovial fluid after 30 days of intervention, and the inflammatory factor IL-1β levels were elevated in both serum and synovial fluid.

**Results:**

On day 10, the model quails exhibited significantly increased serum uric acid levels, indicating hyperuricemia. This condition was accompanied by a decreased uric acid excretion fraction and increased activities of liver uricase, xanthine oxidase (XOD), and adenosine deaminase (ADA). Additionally, there was a significant upregulation of GLUT9 mRNA levels in the kidney, accompanied by a downregulation of renal OAT1, OAT3, and ABCG2 mRNA levels. Although high serum uric acid levels have been observed at this time, no MSU crystals formation or acute inflammation-related manifestations have been noted. On day 20, urate crystals were observed in the kidneys of the model quails, accompanied by elevated serum CRE and BUN levels, alongside evident pathological damage indicative of uric acid nephropathy. Even if high serum uric acid levels persist on day 20, urate crystals and acute inflammation have not yet appeared in synovial fluid, further supporting the notion that crystal deposition is a gradual process rather than triggered by hyperuricemia. By day 30, urate crystals were detected in the synovial fluid of the model quails, and the levels of uric acid and inflammatory cytokine IL-1β in synovial fluid were significant increased, indicating the presence of gouty arthritis. This suggests that uric acid elevation precedes MSU crystal formation, and MSU deposition is a crucial event in the development of gouty arthritis. Furthermore, serum levels of inflammatory cytokines IL-6, TNF-α, and hs-CRP were elevated considerably throughout the modeling process.

**Conclusion:**

This diet-induced quail model successfully recapitulates the progressive pathological stages of human uric acid metabolism disorders, providing a valuable tool for investigating disease mechanisms and evaluating potential therapeutics.

## 1 Introduction

Hyperuricemia is a metabolic disorder characterized by abnormally elevated serum urate levels resulting from excessive uric acid production and/or impaired excretion (1). The prevalence of hyperuricemia is steadily increasing across most countries and regions, particularly in developed and rapidly developing countries. For instance, its prevalence is approximately 20% in the United States, 24.5% in Europe, 26.8% in Japan, and 13.3% in China (2–5). The primary consequences of prolonged hyperuricemia include uric acid nephropathy and gouty arthritis (6, 7). When serum urate exceeds saturation thresholds, MSU crystals can form in the renal tubules and interstitium, leading to kidney damage and potentially inducing renal failure, with a 7–11% increased risk of nephropathy for every 1 mg/dL elevation in serum urate (8, 9). The deposition of MSU crystals in joints and surrounding tissues may result in gouty arthritis, which is clinically manifested by joint erythema, swelling, severe pain, and, in severe cases, joint deformities (10). Approximately 50% of patients with long-term uric acid nephropathy associated with hyperuricemia develop gout (6), highlighting the progressive continuum from hyperuricemia to nephropathy and gout, which aligns with the latest advancements in this research field (5, 11, 12). There is an urgent need for further research into its pathology and the development of effective prevention and treatment strategies. Animal models are essential tools for studying disease pathogenesis and therapeutic interventions. However, an ideal model that recapitulates the sustained and progressive features of uric acid metabolism disorders remains elusive.

Rodents, particularly rats and mice, are commonly employed in laboratory research. Researchers have successfully established models of hyperuricemia, urate nephropathy, and gouty arthritis through various approaches, including chemical induction [e.g., potassium oxonate combined with hypoxanthine for hyperuricemia (13), adenine for urate nephropathy (14) and MSU injection for acute gouty arthritis (15)], dietary induction [e.g., yeast extract combined with fructose water for hyperuricemia (16)] and genetic modification [e.g., uricase-knockout mice (17)]. However, none of these models can simultaneously mimic the progressive pathological transition from hyperuricemia to urate nephropathy and gouty arthritis. Additionally, rodents express uricase, which converts uric acid into the more soluble allantoin, complicating the maintenance of stable hyperuricemia without external manipulation.

Given these limitations, researchers have shifted their focus to avian species. Poultry, including chickens, ducks, quails, and geese, have been observed to exhibit phenomena such as elevated uric acid levels and joint swelling, which are closely associated with the absence of uricase (18). This characteristic provides avian species a distinct advantage over rodent models (e.g., rats and mice) in demonstrating higher and more sustainable uric acid levels when subjected to overnutrition diets (19–22). Our previous research has confirmed that quails, due to their unique traits such as the absence of uricase, small size, and ease of husbandry, are well-suited for studies on hyperuricemia and gouty arthritis (23, 24). In our latest study, we discovered that a nutrient-excess dietary induction method enables quails to consistently exhibit three stages of progressive pathological changes. Clinical evidence and epidemiological studies have shown that diets high in calcium, purines, and fructose are critical risk factors contributing to disorders of uric acid metabolism (25–27).

Consequently, this study aimed to establish a diet-induced animal model with progressive pathological changes associated with uric acid metabolism disorders, which gradually exhibit three stages: hyperuricemia, hyperuricemia combined with uric acid nephropathy, and hyperuricemia combined with gouty arthritis, using quails. This model may provide an innovative approach for researching the pathogenesis of uric acid disorder development and for evaluating the efficacy of potential therapeutic drugs.

## 2 Materials and methods

### 2.1 Animal experiments

All experimental procedures and animal care were approved by the Animal Ethics Committee of the Beijing University of Chinese Medicine (Animal Ethics Approval Number: BUCM-2024070105-3005). Male Defaike quails, aged 25 days, were raised under standard conditions (temperature: 25 ± 2°C, humidity: 50–55%, and a 12-h light cycle). Ventilation was maintained within these standard environmental parameters.

After 3 days of adaptive feeding, the quails were subjected to a 12 h fasting period to collect baseline (0-day) serum samples from the jugular vein for uric acid measurement. Based on their body weights and 0-day serum uric acid levels, the quails were randomly divided into two groups: the control group (Con, *n* = 30), which was fed a standard diet with free access to water, and the model group (Mod, *n* = 40), which received an overnutrition diet supplemented with 20% yeast extract powder and 30% bone extract powder (administered at a rate of 30 g per quail per day), along with restricted water intake (each quail was given 15 mL of 10% fructose-containing water daily). Overnutrition specifically refers to a diet high in purines, calcium, and fructose. Every modeling cycle lasted 10 days. The compositions of the diets are detailed in Table 1.

**Table 1:** Experimental feed proportions and nutritional levels (%).

Items	Diet composition	The control group	The model group
Feed ratio	Corn	55.00	26.00
	Soybean meal	33.00	15.60
	Fish meal	2.00	1.00
	Stone powder	5.00	2.40
	Bovine bone meal	–	30.00
	Yeast extract powder	–	20.00
	premix	5.00	5.00
	Total	100.00	100.00
Nutrient levels	CP	21.70	29.50
	Ca	1.00	7.80
	AP	0.50	3.73

The premix provides the following nutrients per kg diets: VA 200,000 IU, VD3 60,000 IU, VE 380 mg, VK3 56 mg, VB156 mg, VB2 200 mg, VB6 60 mg, VB12 300 μg, biotin 3 mg, niacin 600 mg, pantothenic acid 240 mg, folic acid 30 mg, Cu 0.2 g, Fe 1.8 g, Mn 1.4 g, Zn 1.1 g, Se 6 mg, I 8.5 mg, P 15–60 g, Ca 100–250 g, NaCl 50–150 g, amino acids 28 g.

Blood samples were collected every 10 days. Following a 12-h fasting period, 0.8 mL of blood was drawn from the right jugular vein of quails using a sterile syringe. The blood sample was then transferred into an Eppendorf tube. After being left at room temperature for 2 h, the sample was centrifuged at 3,000 r/min for 10 min. The supernatant serum was subsequently aspirated and reserved for the subsequent determination of serum uric acid, XOD, ADA, and BUN levels (Nanjing Jiancheng Bioengineering Institute, China). After blood collection, some quails were sacrificed in stages to obtain liver, kidney, ankle joint, and synovial tissue samples for subsequent histopathological examination. Additionally, the left ankle joint cavity of each quail was rinsed with 0.2 mL of saline to collect fluid for analyses of uric acid and inflammatory cytokines. The right ankle joint cavity was rinsed with 0.2 mL of anhydrous ethanol to collect fluid for polarized light microscopy of MSU. The entire experiment was conducted over a period of 30 days.

Based on clinical diagnostic markers and the characteristics observed in animal experiments, the success criteria for evaluating the model are as follows: (1) Hyperuricemia, with an intervention for 10 days. The key indicators include a significant increase in serum uric acid levels, while there is no notable deposition of MSU crystals in the kidneys or synovial fluid. (2) Hyperuricemia combined with uric acid nephropathy, with an intervention duration of 20 days. The key indicators are the deposition of renal MSU crystals, while auxiliary indicators include persistently elevated serum uric acid levels, significant increase in renal function-related parameters (such as CRE, and BUN), and pathological changes in renal tissue. (3) Hyperuricemia combined with gouty arthritis, with an intervention duration of 30 days. The key indicators are the deposition of MSU crystals in the synovial fluid and an elevation of the serum inflammatory cytokine IL-1β, while auxiliary indicators include persistently elevated serum uric acid levels.

### 2.2 Detection of uric acid and creatinine levels in the feces-urine mixture

Metabolic cages were employed every 10 days to collect the feces-urine mixture from quails following a 12 h fasting period, during which they had free access to water. A 0.2 g sample of the feces-urine mixture was weighed, combined with 1.8 mL of saline, vortexed until homogeneous, and then centrifuged to obtain the supernatant. The supernatant was collected for the subsequent detection of uric acid and creatinine (Nanjing Jiancheng Bioengineering Institute, China), following the instructions provided with the corresponding kits.

### 2.3 Calculation of uric acid excretion fraction

The uric acid excretion fraction of quails was calculated at 10, 20, and 30 d using the following formula: Uric acid excretion fraction (%) = (Fecal-urinary mixture uric acid * Serum creatinine/Serum uric acid * Fecal-urinary mixture creatinine) * 100%.

### 2.4 Detection of inflammatory cytokines

Enzyme-linked immunosorbent assay kits were utilized to measure levels of inflammatory factors, including Interleukin-1β (IL-1β), Interleukin-6 (IL-6), Hypersensitive C-reactive protein (hs-CRP), and Tumor Necrosis Factor-α (TNF-α) (Jianglai Biology, Shanghai) in serum on days 10, 20, and 30, as well as IL-1β level in synovial fluid. Neither serum nor synovial fluid samples were diluted, and all procedures were conducted in strict accordance with the reagent instructions.

### 2.5 Observation of MSU deposition in the kidney and joint using polarized light microscopy

Following fixation in anhydrous ethanol overnight, the kidneys underwent dehydration, dewaxing, paraffin embedding, sectioning, dewaxing, and mounting (note: the kidney tissue should not come into contact with water throughout the experimental process). An optical microscope equipped with a polarized filter (Nikon ECLIPSE E200) was used to observe MSU in the kidneys. In addition, wash the joint cavities of quails with 0.2 mL of physiological saline and collect synovial fluid from each group. Polarized light microscopy serves as the gold standard for diagnosing gout (28), and the crystal morphology features conform to international consensus (29).

### 2.6 Histological analysis

Liver, kidney, and joint tissues were collected and fixed in 4% paraformaldehyde (Servicebio, China) at room temperature for 48 h. The joint tissues underwent decalcification in a 10% EDTA solution (Aqlabtech, China) at room temperature for 45 days. Subsequently, the tissues were embedded in paraffin, and paraffin sections (3 μm thick) were prepared, followed by dewaxing and dehydration. Finally, the sections were stained with hematoxylin and eosin (H&E). Synovial hyperplasia and inflammation were evaluated using the Krenn scoring system (23) on H&E-stained sections. This system semi-quantitatively assesses three parameters: the synovial lining cell layer, stromal cell density, and inflammatory infiltrate using a scale of 0–3 (0 = none, 1 = slight, 2 = moderate, 3 = strong) (30). The individual subscores were summed to yield a total Krenn score ranging from 0 to 9, with higher scores indicating more severe synovial proliferation and inflammation.

### 2.7 Reverse transcription quantitative polymerase chain reaction (RT-qPCR) analysis

Total RNA of kidney tissue was extracted with TRIzol reagent (Thermo Fisher Scientific, USA) and quantified using a NanoDrop ONEc spectrophotometer (Thermo Fisher Scientific, USA). Then, the total RNA was reverse transcribed into cDNA using the RevertAid First Strand cDNA Synthesis Kit (Thermo Fisher Scientific, USA). cDNA amplification was performed using PowerUp™ Green Master Mix (Thermo Fisher Scientific, USA) on a CFX96 Real-Time PCR System (Bio-Rad, USA). The relative gene expression levels were determined using the 2^−ΔΔCT^ method. Specific primers were purchased from Beijing Bomaide Gene Technology Co., Ltd., and their sequences are listed in Table 2.

**Table 2:** Quail related gene specific primer sequences.

Gene name	Upper/lower primer	Sequence (5′–3′)
GLUT9	Forward primer	GCATCATTCTGCATTGGACC
	Reverse primer	AAGTTGGAGAGCCAGTTGAC
OAT1	Forward primer	CTGCGCCTACATCTTCACCG
	Reverse primer	CCACGTCCTCCACAGTTTCG
OAT3	Forward primer	TCGCCTACGCCGTCCCACA
	Reverse primer	TTCCTTCCCCGCCAGCACC
ABCG2	Forward primer	CAGCAAGCAAGGAAGATCAC
	Reverse primer	GGCTGGAGTTGAGATACTTC
β-actin	Forward primer	GATGAAGCCCAGAGCAAAAGA
	Reverse primer	ACCAGAGGCATACAGGGACAG

GLUT9: Solute Carrier Family 2, Facilitated Glucose Transporter Member 9; OAT1: Solute Carrier Family 22 Member 6; OAT3: Solute Carrier Family 22 Member 8; ABCG2: ATP-binding Cassette, Sub-family G (WHITE), Member 2.

### 2.8 Statistical analysis

Statistical analyses and graphical representations were performed using GraphPad Prism 9 (GraphPad Software, USA). Data are presented as mean ± standard deviation (SD). Initially, normality and homogeneity of variance tests were performed. For two-group data that met the assumptions of normal distribution and homogeneity of variance, an unpaired t-test was utilized. In cases where the two-group data did not conform a normal distribution, the Mann–Whitney U test was employed. When the two-group data adhered to normality but exhibited unequal variances, a t-test with Welch’s correction was applied. *p* < 0.05 was considered statistically significant.

## 3 Results

### 3.1 The elevated serum uric acid levels and impaired uric acid excretion in diet-induced quail model persisted throughout the experimental period

**Figure 1:**
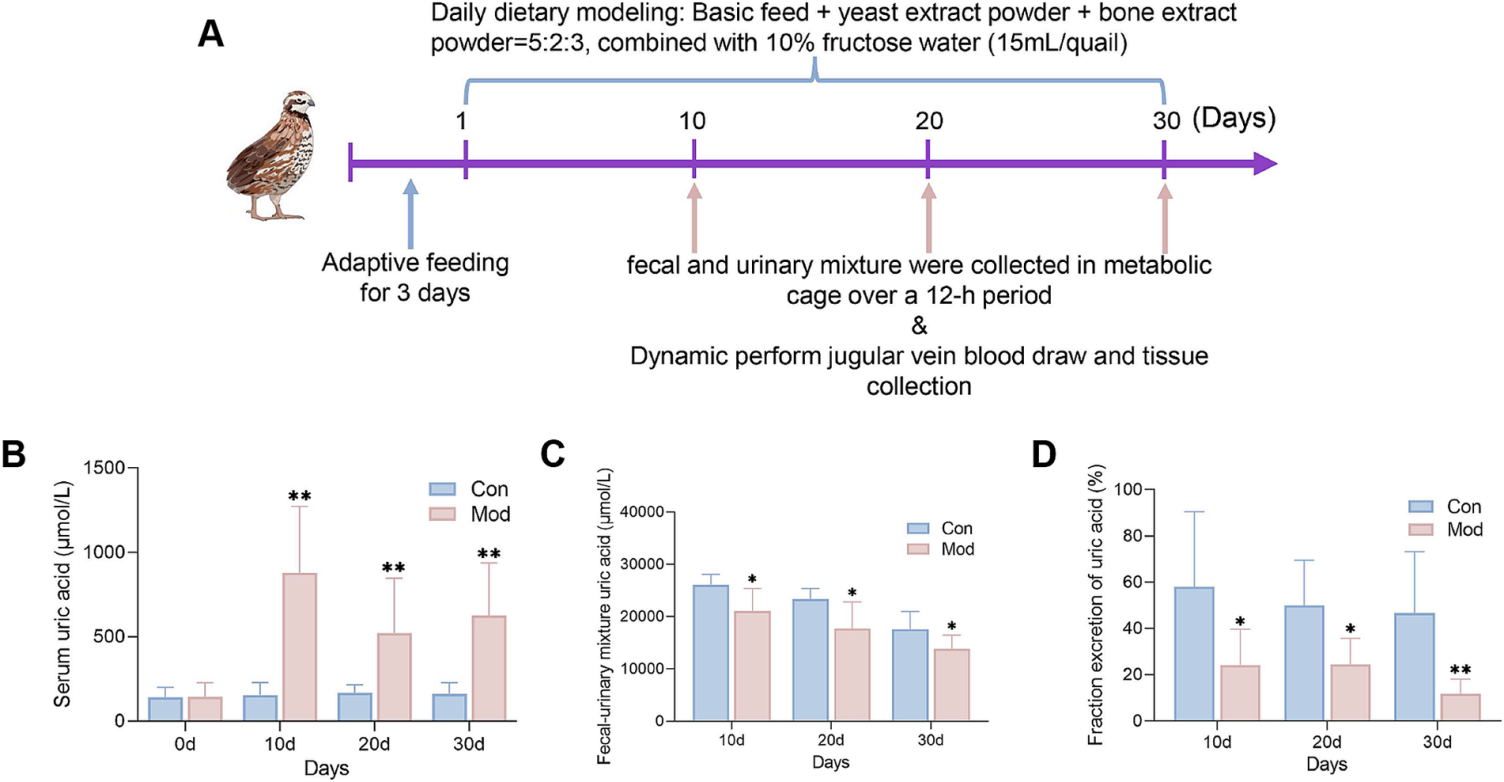
Levels of serum uric acid and uric acid excretion during the experimental period. **(A)** Schematic diagram of the study protocol. **(B)** Serum uric acid level (On day 10, *n* = 30 per group; On day 20, *n* = 20 in the Con group and *n* = 13 in the Mod group; On day 30, *n* = 10 in the Con group and *n* = 7 in the Mod group). **(C)** Uric acid levels in the fecal-urinary mixture (*n* = 7 per group). **(D)** 12 h uric acid excretion fractions (*n* = 7 per group). Data are presented as mean ± SD. All data are expressed as mean ± SD. Statistical significance is indicated as ^*^p < 0.05, ^**^p < 0.01.

The entire experimental period lasted for 30 d (Figure 1A). Serum uric acid levels serve as key indicators of hyperuricemia. The results showed that compared to the Con group, serum uric acid levels in the Mod group of quails significantly increased from day 10 and persisted until day 30 (Figure 1B). Fecal-urinary mixture uric acid levels and the 12 h uric acid excretion fraction are effective measures for evaluating uric acid excretion (20). Compared to the Con group, the uric acid levels in the fecal-urinary mixture and the 12 h fraction excretion of uric acid in the Mod group were significantly reduced at all time points (Figures 1C,D). These findings suggest that hyperuricemia induced by nutritional excess can be established by day 10 and that persists until the end of the experiment.

### 3.2 The abnormal expression levels of purine metabolizing enzymes and uric acid transporters in diet-induced quail model of hyperuricemia stage

The production and excretion of uric acid in the body depend on purine metabolizing enzymes and the uric acid transporter protein (31). XOD and ADA are key rate-limiting enzymes that convert endogenous purines into uric acid (32, 33). GLUT9 serves as a crucial uric acid reabsorption transporter, facilitating the reabsorption of uric acid into the bloodstream (34). OAT1, OAT3, and ABCG2 are uric acid secretion proteins that enhance the excretion of serum uric acid via urine (35). We measured the activities of XOD and ADA in serum, as well as the mRNA expression levels of GLUT9, OAT1, OAT3, and ABCG2 in the kidneys on day 10.

**Figure 2:**
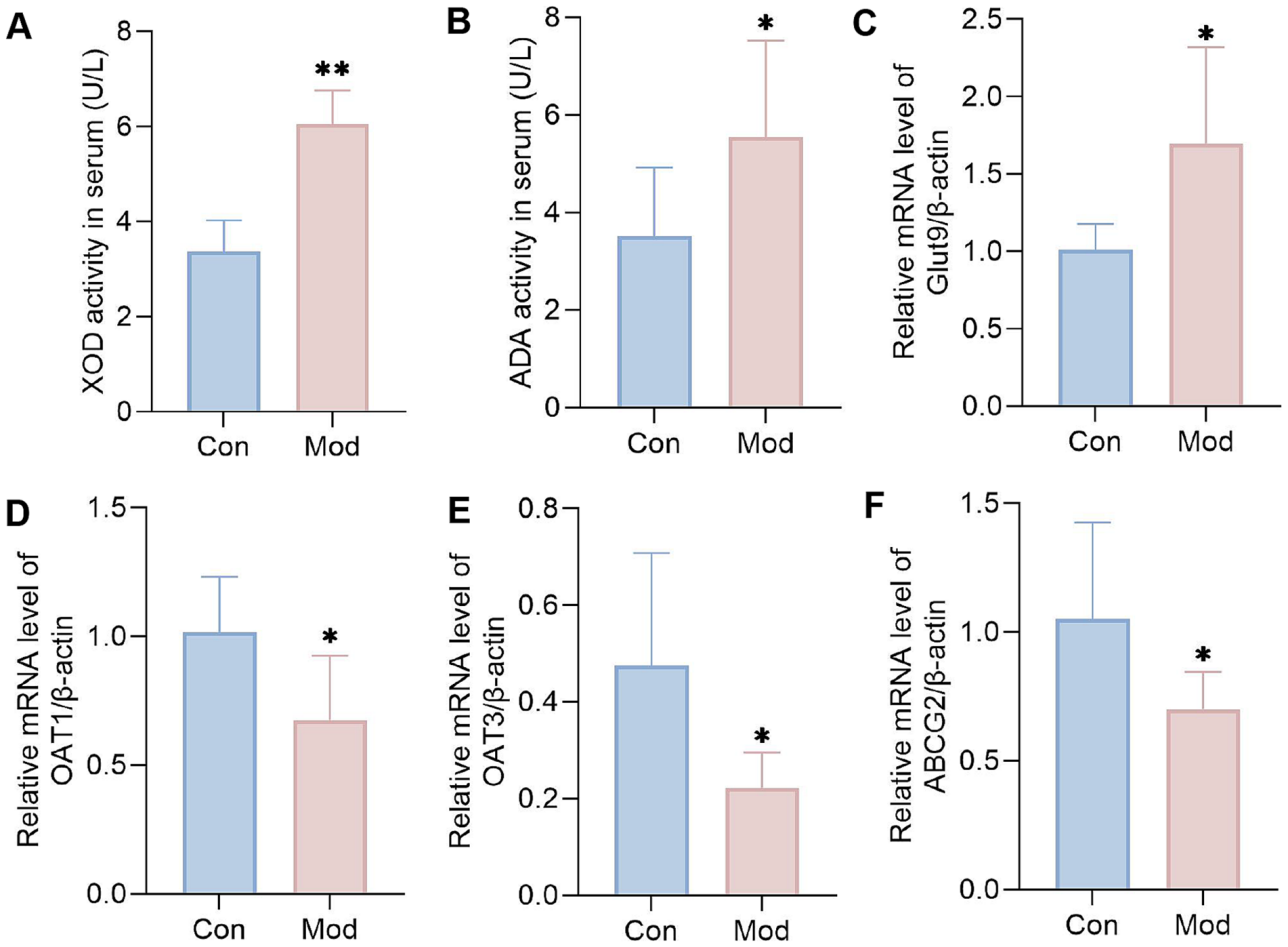
Levels of purine metabolizing enzymes and uric acid transporters on day 10. **(A)** XOD activity in serum (*n* = 10 per group). **(B)** ADA activity in serum (*n* = 10 per group). Bar diagram showing the relative expression levels of **(C)** GLUT9 mRNA, **(D)** OAT1 mRNA, **(E)** OAT3 mRNA, and **(F)** ABCG2 mRNA, *n* = 6 per group. All data are expressed as mean ± SD. Statistical significance is indicated as ^*^p < 0.05, ^**^p < 0.01.

The results showed that, compared to the Con group, the serum activities of XOD and ADA in the Mod group were significantly elevated (Figures 2A,B). Furthermore, the renal mRNA expression level of GLUT9 was increased considerably, while the mRNA expression levels of renal OAT1, OAT3, and ABCG2 were significantly decreased (Figures 2C–F). This result proves that the key pathological mechanism underlying diet-induced hyperuricemia is associated with the abnormal expression of purine metabolizing enzymes and uric acid transporters. It also indicates that the hyperuricemia quail model caused by overnutrition can be established on the 10th day.

### 3.3 The observation of MSU deposition in the kidney and renal function of diet-induced quail model during the experimental period

**Figure 3:**
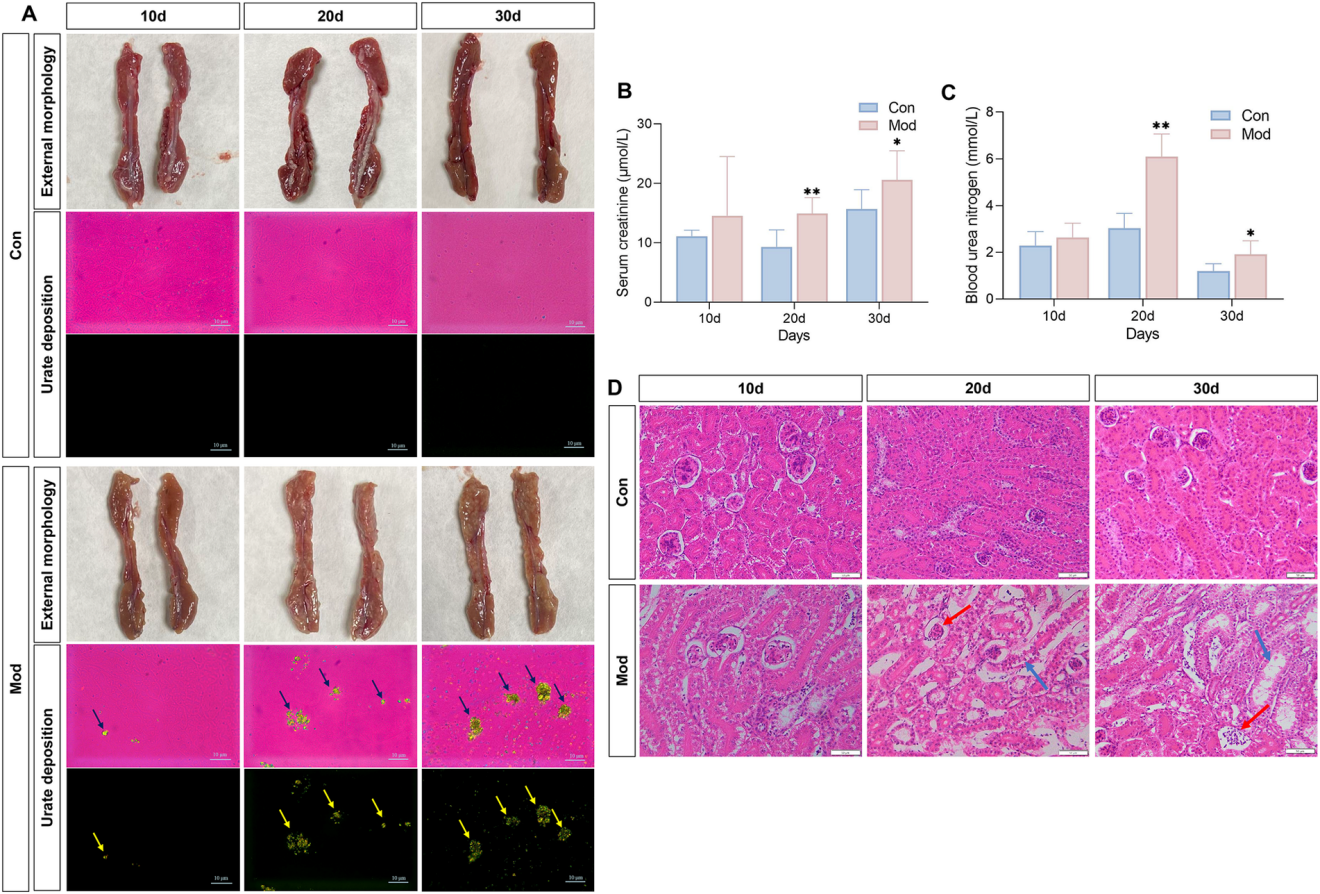
Overnutrition leads to renal MSU deposition, renal dysfunction, and renal pathological damage. **(A)** External morphology of quail kidneys and renal MSU deposition under polarized light on days 10, 20, and 30 (red background indicates bright field and black background indicates dark field). **(B)** Serum creatinine level. **(C)** Blood urea nitrogen levels. **(D)** Pathological image of renal H&E staining. *n* = 7 per group. All data are expressed as mean ± SD. Statistical significance is indicated as ^*^p < 0.05, ^**^p < 0.01.

Persistently elevated serum uric acid levels can overwhelm renal excretion, resulting in uric acid supersaturation. This condition leads to the deposition of MSU in the kidneys, exacerbating renal damage and creating a vicious cycle of abnormal uric acid metabolism (36). During the experiment, the kidneys in the Con group exhibited a standard deep red color, characterized by a smooth surface and the absence of apparent abnormalities. In contrast, the kidneys in the Mod group appeared slightly bloodless on day 10 and became increasingly pale and bloodless on days 20 and 30, with a surface exhibiting white, dot-like changes that formed a “mottled kidney” (Figure 3A). Similarly, we observed a few MSU deposits in the kidneys of some quails in the Mod group under polarized light microscopy. However, significant MSU deposition was evident in the kidneys of all quails in the Mod group on days 20 and 30 (Figure 3A).

The serum levels of Cre and BUN are sensitive indicators for assessing renal function (37–39). Compared to the Con group, the Mod group exhibited a significant increase in serum Cre and BUN levels on days 20 and 30 (Figures 3B,C). Additionally, we examined the histopathological changes in the kidney tissue of quails using H&E staining. On days 10, 20 and 30, the Con group exhibited normal kidney structure with no signs of hyperplasia or atrophy in the glomeruli or tubules. In contrast, starting from day 20, the Mod group exhibited glomerular atrophy, enlarged glomerular capsules, and vacuolar degeneration in tubules compared to the Con group (Figure 3D).

Collectively, the evident MSU deposition in the kidneys, combined with the impaired renal function observed in the quails of the Mod group, indicates that uric acid nephropathy induced by nutritional excess can be established by day 20.

### 3.4 The observation of MSU deposition and inflammation in the joint of diet-induced quail model during the experimental period

**Figure 4:**
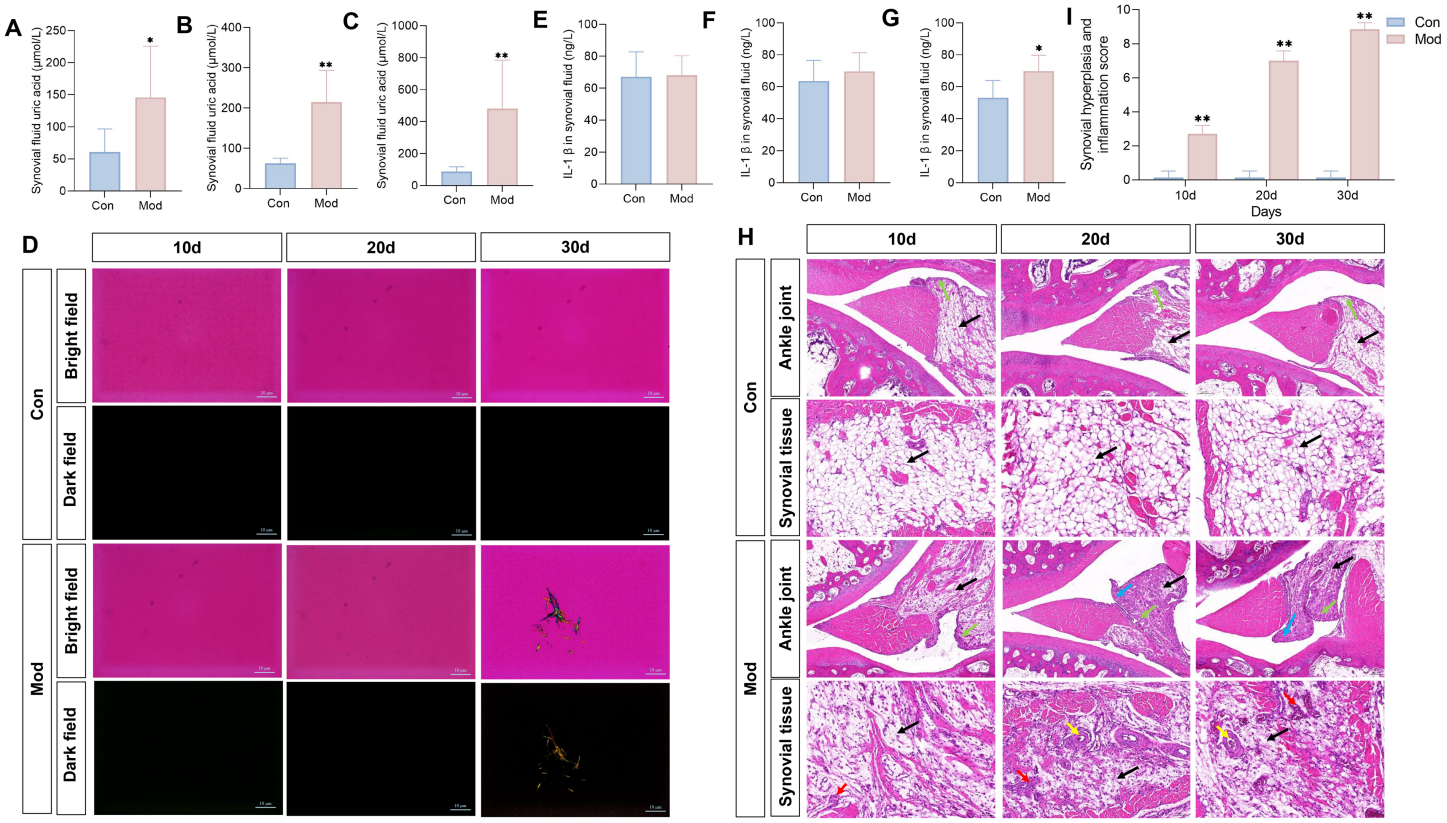
Overnutrition leads to MSU deposition in quail synovial fluid and joint pathological damage. Synovial fluid uric acid in quails on day 10 **(A)**, day 20 **(B)**, and day 30 **(C)**, *n* = 7 per group. **(D)** Observation of MSU crystals in the synovial fluid of quails on days 10, 20, and 30, under polarized light. Levels of IL-1β in the synovial fluid of quails on day 10 **(E)**, day 20 **(F)**, and day 30 **(G)**. *n* = 10 on day 10 per group, *n* = 7 on day 20 and 30 per group. **(H)** H&E staining of quail ankle joints. **(I)** Synovial hyperplasia and inflammation score. Blue arrow represents synovial hyperplasia toward the joint cavity, the green arrow represents the lining layer, the black arrow represents the cell matrix, the red arrow represents inflammatory cell infiltration, and the yellow arrow represents the formation of vascular opacities. *n* = 7 per group. All data are expressed as mean ± SD. Statistical significance is indicated as ^*^p < 0.05, ^**^p < 0.01.

To investigate MSU deposition in the joints of quails in the Mod group, we examined uric acid levels in the synovial fluid. We found a consistent and significant increase in synovial fluid uric acid levels in the Mod group on days 10, 20, and 30 (Figures 4A–C). According to the clinically established gold standard for diagnosing gouty arthritis, which relies on the presence of MSU in synovial fluid aspirates (40), we found no MSU deposition in the synovial fluid of the Mod group quails on days 10 and 20; needle-like crystals appeared only on day 30 (Figure 4D). Additionally, only on day 30, the IL-β levels in the synovial fluid on the Mod group quails significantly increased (Figures 4E–G). Histopathological observations of the ankle joint tissue revealed that on days 10, 20, and 30, the ankle joint structure of quails in the Con group was well-defined, with regularly arranged synovial tissue. In contrast, the quails in the Mod group exhibited dilated and congested blood vessels, inflammatory cell infiltration, and synovial hyperplasia that extended into the joint cavity. These conditions worsened over time, particularly by day 30 (Figure 4H). The synovitis score showed that the model group had significantly higher scores compared to the normal group, with the highest scores recorded on the 30th day (Figure 4I).

Collectively, the evident MSU deposition in the synovial fluid, along with the inflammation and histopathological impairment observed in the joints of quails in the Mod group, indicates that gout arthritis induced by nutritional excess can be established by day 30.

### 3.5 The observation of serum inflammatory cytokines in the diet-induced quail model during the experimental period

**Figure 5:**
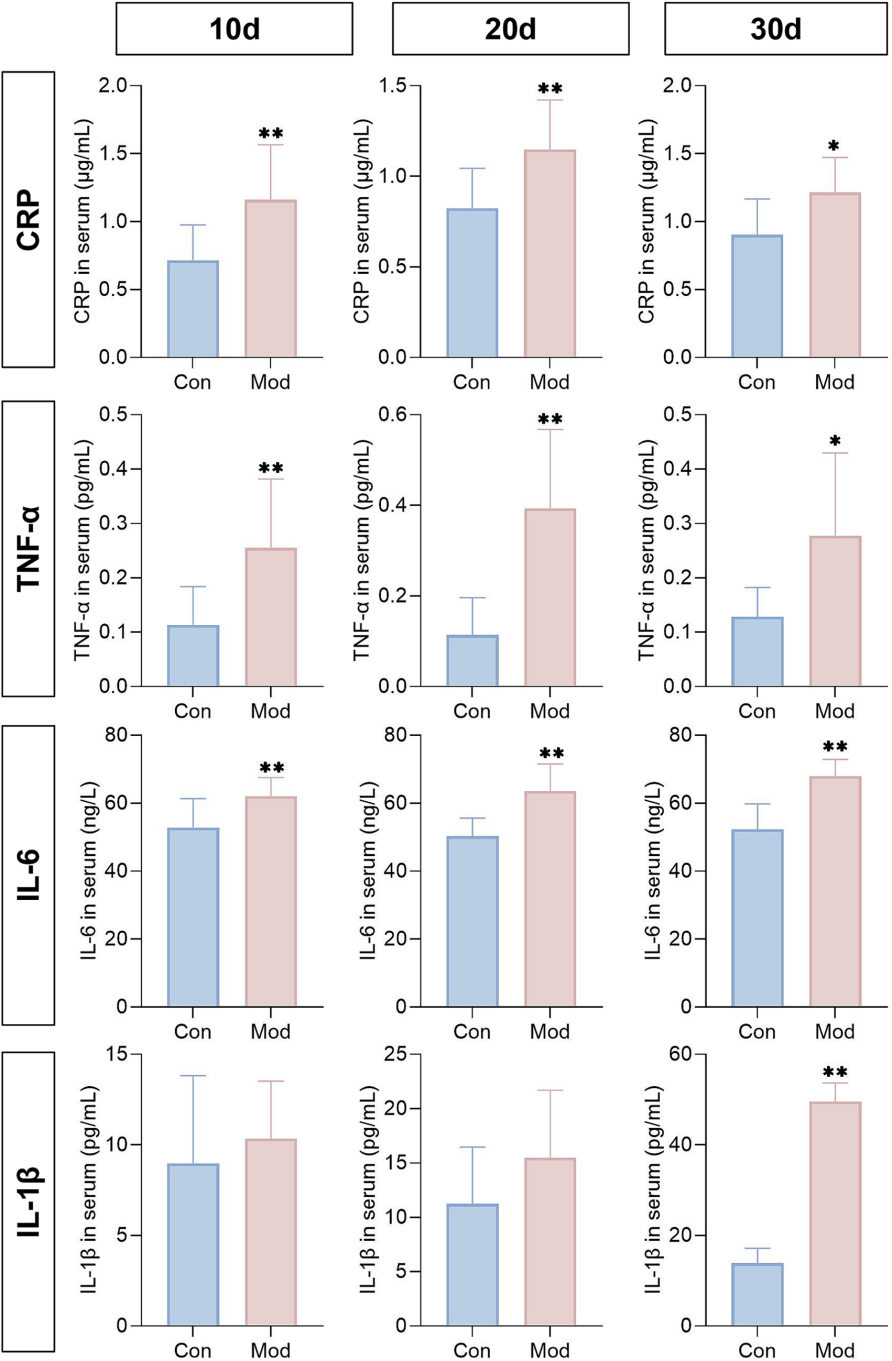
Levels of inflammatory cytokines during the experimental period. The concentrations of IL-6, hs-CRP, TNF-α, and IL-1β in quail serum on days 10, 20, and 30 were determined. *n* = 10 on day 10 and 20, *n* = 10 in the con group and *n* = 7 in the mod group on day 30. All data are expressed as mean ± SD. Statistical significance is indicated as ^*^p < 0.05, ^**^p < 0.01.

According to reports that both soluble uric acid and MSU can promote inflammation (41, 42), we dynamically measured the levels of serum inflammatory cytokines in experimental quails, including IL-6, hs-CRP, TNF-α, and IL-1β. The results showed that on days 10, 20, and 30, the Mod group exhibited significantly elevated levels of hs-CRP, TNF-α, and IL-6 in quail serum compared with the Con group, with no significant change in IL-1β. By day 30, the model group showed a substantial increase in serum IL-1β levels (Figure 5). This indicates that the overall pathological state of the quail model exhibits a characteristic progression from low-grade inflammation to acute inflammation. This finding aligns with clinical reports of increased levels of hs-CRP, TNF-α, and IL-6 in the serum of patients with hyperuricemia and uric acid nephropathy, as well as elevated IL-1β expression in the serum of patients with gouty arthritis (43, 44).

## 4 Discussion

### 4.1 Quails serve as the ideal animal for the study of uric acid metabolism disorders

The quail, classified under the class Aves, order Galliformes, family Phasianidae, and genus Coturnix, is scientifically designated as *Coturnix japonica*. In the late 20th century, quails became significant subjects of research due to their practical advantages, including small size, high disease resistance, and short generational intervals of approximately 3 months. These characteristics make them particularly suitable for genetic and toxicological studies. The completion of their genome sequencing has further underscored their importance in contemporary biomedical research, particularly in developmental studies, environmental toxicology, and human disease modeling (45).

In the early 1990s, our research group was among the first to systematically utilize quail in studies of hyperuricemia, capitalizing on their unique biological characteristic: a natural deficiency of uricase, similar to that found in humans (46). This physiological trait increases the risk of hyperuricemia and related diseases, such as gout, in both humans and quail. Through our investigations, we developed a modeling approach that combines a high-calcium, high-purine diet with fructose water supplementation. This method maintains stable elevated serum urate levels in quail and induces MSU crystal deposition in renal tissues and joint synovial fluid. Consequently, we established a comprehensive progressive animal model encompassing three pathological stages: hyperuricemia, hyperuricemia combined with urate nephropathy, and hyperuricemia combined with gouty arthritis. This quail model naturally recapitulates the progressive pathological changes associated with human urate metabolism disorders, thereby serving as a valuable complement to rodent models. No other animal model can consistently demonstrate all three pathological stages within a single system despite rodents being the most commonly used laboratory animals.

Rodent models (rats and mice) naturally express urate oxidase, necessitating continuous administration of urate oxidase inhibitors (e.g., potassium oxonate) to induce and maintain hyperuricemia. However, this method cannot spontaneously induce gouty arthritis unless supplemented with exogenous MSU crystal injections into joint cavities (22, 47). Such injections fail to replicate the systemic pathological state associated with elevated serum urate levels. To effectively model gouty arthritis under hyperuricemic conditions, additional administration of urate oxidase inhibitors is required. These interventions not only diverge significantly from human urate metabolism pathways but also raise concerns regarding model stability and the potential for nonspecific organ damage due to exogenous compounds (13). Although urate oxidase-deficient (Uox^−^/^−^) rodent models have provided valuable insights, some limitations remain. For instance, Uox^−^/^−^ models primarily simulate isolated hyperuricemia (HUA) without facilitating the progressive development of gouty arthritis. Uricase knockout induces systemic effects that extend beyond urate metabolism, including developmental, metabolic, and cardiovascular abnormalities (48), thereby introducing experimental confounders. The substantial costs associated with maintaining Uox^−^/^−^ colonies pose significant barriers to large-scale studies (48, 49). Beyond rodent models, researchers have also utilized primates and zebrafish for urate metabolism studies. While primates theoretically represent ideal models due to their nearly identical urate metabolic pathways to humans, their use is constrained by ethical regulations, high costs, and technical challenges. Zebrafish models are still in the preliminary exploration stages (50). In contrast, quails exhibit a natural deficiency in uricase akin to humans while providing practical advantages such as low maintenance costs and ease of husbandry, making them uniquely suited for the study of urate metabolism disorders.

### 4.2 Dietary overnutrition is the primary clinical cause of uric acid metabolism disorders and can serve as an inducer in animal models

Uric acid metabolism is influenced by various factors, such as genetics, race, age, gender, geographical environment, and diet (7). Among these, alterations in dietary structure have emerged as a significant trigger for clinical hyperuricemia and gout (25, 51). For example, a case–control crossover study demonstrated that exposure to purine-rich foods, such as red meat, seafood, and sugary foods, increased the risk of recurrent gout attacks by fivefold (52). Additionally, a longitudinal study showed that the consumption of sugary drinks elevates the risk of hyperuricemia in adults (53). Conversely, dietary patterns such as the Mediterranean Diet, which is abundant in plant proteins, whole grains, olive oil, and fish, as well as the Dietary Approaches to Stop Hypertension Diet, rich in whole grains, fruits, vegetables, and low-fat dairy products, along with a low-purine diet that avoids shellfish, organ meats, and alcoholic beverages, are associated with a reduced risk of uric acid metabolism disorders (54, 55). Both the ACR 2020 and EULAR 2016 guidelines recommend the conditional limitation of high-purine foods (e.g., red meat, organ meats, seafood) and fructose intake, regardless of disease activity (56, 57). This underscores the well-established role of purine-rich diets in the promotion of uric acid dysregulation. Excessive intake of high-purine foods promotes *de novo* purine synthesis and XOD activity, thereby increasing uric acid production (58). The phosphorylation of fructose results in ATP depletion, augmenting the compensatory effect on purine synthesis (59). In studies investigating the pathogenesis and intervention strategies of hyperuricemia, many studies have successfully established hyperuricemia animal models through the administration of high-purine diets and fructose induction. For instance, researchers induced hyperuricemia in mice by administering yeast extract combined with a 10% fructose solution (60). Our previous research also found that the serum uric acid levels in quails consistently increased following prolonged intake of a high-purine diet (61).

In addition to excessive uric acid production, disorders in uric acid excretion represent a significant factor in abnormal uric acid metabolism, with renal excretion being particularly critical. As the primary pathway for uric acid clearance in the body, the kidneys are responsible for approximately 2/3 of uric acid excretion (62). Clinical research reports that over 90% of patients with primary fall into the category of renal excretion disorder (63). Research has reported that prolonged high-calcium diets can impair renal function and inhibit uric acid excretion (64). The influx of substantial calcium ions into the bloodstream tends to bind with uric acid, which can subsequently impair the kidney (65, 66). Research has shown that the prolonged administration of high-calcium feed to poultry, including chickens and quails, may result in gout affecting the internal organs and joints (67). Additionally, insufficient water intake is another factor contributing to abnormal renal function, as optimal renal function relies on adequate hydration (68). When the body experiences relative dehydration, urine concentration increases, and both fecal and urinary excretion decrease. This reduction inhibits uric acid excretion, exacerbating serum uric acid levels and potentially leading to MSU deposits in the kidneys, which can further impair renal function (69). Therefore, the 2020 American Society of Nephrology Clinical Practice Guidelines recommends that healthy adults should consume between 2.5 and 3.7 L of water daily for males and between 2.0 and 2.7 L for females (70).

Based on the analysis of the clinical factors associated with disorders of uric acid metabolism, this study successfully established a stable animal model of abnormal uric acid metabolism in quails. This model, which can be maintained for 30 days, was induced by combining a high-purine, high-calcium, and high-concentration fructose diet with restricted water intake, demonstrating significant clinical relevance.

### 4.3 Characteristics of the progressive pathological alterations associated with disordered uric acid metabolism in the quail model

Hyperuricemia, uric acid nephropathy, and gouty arthritis are prevalent diseases associated with disorders of uric acid metabolism. Currently, the development of experimental animal models for these conditions has become relatively advanced. Hyperuricemia can be induced in rat or mouse models through gavage or intraperitoneal injection of potassium oxonate, while gouty arthritis models can be established via intra-articular injection of MSU (22, 47). However, clinical research has revealed the progressive nature of uric acid metabolism disorders (5). There is a growing focus on studying the pathological mechanisms underlying the transition from hyperuricemia to uric acid nephropathy and gouty arthritis, as well as on the development of pharmacological interventions aimed at comprehensively regulating these disorders. Existing animal models are inadequate for research purposes, underscoring the urgent need for a model that accurately simulates the progressive characteristics of uric acid metabolism disorders. Our research has provided an innovative experimental animal model to fulfill this requirement.

In this study, on day 10, we observed significantly elevated serum uric acid levels in the model quail without apparent renal damage or MSU deposition in the kidneys and joints, thereby mimicking the hyperuricemia stage. By day 20, all euthanized model quails exhibited MSU deposition in the kidneys, accompanied by renal dysfunction and structural abnormalities, indicating the progression to uric acid nephropathy. Despite high UA levels on the 10th and 20th day, MSU and acute inflammation did not immediately appear. By day 30, 71% of the model quails displayed MSU deposition in the synovial fluid, along with increased uric acid levels and significant acute inflammatory response (IL-1β significantly increased), effectively simulating the stage of gouty arthritis. These findings suggest that the quail model accurately reflects the natural course of uric acid metabolism disorders. This also indicates that uric acid elevation precedes crystal deposition, which is a key event in the development of gouty arthritis. However, the reported incidences of uric acid nephropathy and gouty arthritis in this study are case-based, and individual variations among quails and limitations in sample size may introduce deviations.

In terms of pathological mechanisms, the notably increased mRNA expression levels of GLUT9, a uric acid reabsorption transporter, in the kidneys, along with the significantly elevated activities of XOD and ADA in the serum, as well as the significantly reduced mRNA expression levels of OAT1 and OAT3 in the kidneys, which are the primary uric acid secretion proteins, constitute the core pathological mechanism of hyperuricemia in our quail models. GLUT9 is a uric acid reabsorption transporter located on both the apical and basolateral membranes of renal proximal tubular cells. It can reabsorb uric acid from the renal tubules and transport it to the bloodstream, thereby increasing uric acid levels in the body (71). Its genetic polymorphism has been confirmed to be directly associated with hyperuricemia in humans (72). OAT1/3 and ABCG2 are uric acid secretion transporters, expressed in the basolateral and apical membranes of the renal proximal tubules, respectively (73, 74). They are responsible for transporting uric acid from the blood to the renal tubules for secretion into the urine. Reduced expression of OAT1/3 significantly inhibits uric acid excretion (75). XOD and ADA serve as rate-limiting enzymes in the purine nucleoside metabolism pathway, directly or indirectly regulating uric acid levels in the body. XOD can catalyze the conversion of hypoxanthine to xanthine, ultimately producing uric acid (76). ADA indirectly promotes uric acid production by increasing purine degradation substrates (77). The increased activity of both enzymes leads to an excess synthesis of uric acid. The formation of MSU is crucial in the progression from hyperuricemia to uric acid nephropathy or gouty arthritis (36). Polarized light microscopy revealed the presence of yellow-green, nearly round urate crystals in the kidneys of model group quails. MSU exhibits various morphologies influenced by factors such as temperature, ion concentration, and pH. These morphologies include needle-like structures, arcuate aggregates, sea urchin-like aggregates, and beach ball-like forms (66). Among these, the “beach ball-like” MSU crystal is considered an essential metastable precursor in the morphological evolution of urate crystal (66). This observation aligns closely with the MSU crystal morphology observed in quail kidneys in our study. Renal MSU crystals activate the NLRP3 inflammasome in monocytes and macrophages, triggering the extracellular release of inflammatory cytokines and subsequent recruitment of leukocytes, thereby promoting inflammation (78). However, the pathological mechanisms through which ‘beach ball-like’ MSU crystals induce inflammatory activation or tissue damage remain unclear and warrant further investigation. Research reported that the pathogenesis of gouty arthritis might be linked to the morphological transformation of MSU from “beach ball-like” to “needle-like” forms (79), as evidenced by the presence of needle-like MSU crystals in the synovial fluid of the model quails on the 30th day of this study. Studies demonstrate that needle-shaped MSU crystals physically pierce cell membranes due to their sharp structure, leading to lysosomal rupture and the activation of the NLRP3 inflammasome, which subsequently induced acute gouty arthritis (80). Additionally, studies have reported that persistent low-grade inflammation may be a critical factor in promoting uric acid metabolic disorders and exacerbating MSU deposition, as inflammation can intensify tissue and organ damage (81–84). Clinical data indicate that patients with hyperuricemia or uric acid nephropathy exhibit significantly higher levels of low-grade inflammatory markers, such as hs-CRP, TNF-α, and IL-6, in their serum compared to healthy individuals (43). In our model quails, we also observed this characteristic, finding significantly elevated levels of low-grade inflammatory factors on days 10, 20, and 30. At the same time, the deposition of MSU crystals in the kidneys increases over time, indicating that low-grade inflammation may serve as a significant promoter of MSU deposition. In the model group, serum IL-1β levels in quails increased only on day 30 coinciding with the onset of gouty arthritis. During this period, needle-shaped MSU crystals were observed in the synovial fluid, which were absent on days 10 and 20. This temporal characteristic aligns well with the pathological process of the ‘crystal inflammation’ cascade observed during acute gout attacks in clinical practice. Additionally, these findings suggest that IL-1β could serve as a specific biomarker for acute gout inflammation. Collectively, this quail model addresses the limitations of traditional single-phenotype models, which are unable to simulate the progression of uric acid metabolism disorders. It offers a valuable animal model for in-depth exploration of the pathological mechanisms and the evaluation of potential therapeutic approaches, providing significant practical value.

Indeed, this quail model presents specific challenges. First, the basal metabolism in quails differ from humans, which may limiting the model’s capacity to fully recapitulate the spectrum of human urate metabolism disorders. Future studies should systematically validate the clinical relevance of this model to enhance its reliability and utility. Second, the absence of quail-specific research reagents, particularly antibodies, poses significant challenges for mechanistic investigations, highlighting the necessity for the development of avian-specific biological tools. Third, as an exploratory model, it requires more comprehensive validation, including the characterization of intestinal urate transporters and gut microbiota dynamics, systematic evaluation of MSU deposition patterns across multiple organs, and investigation of pathological targets, mechanisms, and genetic signatures associated with progressive hyperuricemia in this model.

## 5 Conclusion

In conclusion, we successfully established a quail model that comprehensively simulates the progressive pathological changes associated with uric acid metabolism disorders. This model compasses the pathological progression from hyperuricemia to uric acid nephropathy and gouty arthritis, achieved through a dietary induction method involving the daily feeding of quails with a high-calcium, high-purine diet alongside a quantitative 10% fructose solution. This innovative animal model serves as a valuable tool for investigating the pathogenesis of uric acid disorders and can be employed in drug efficacy evaluations, safety studies, and the development of new therapeutic strategies for regulating diseases related to uric acid disorders.

## Data Availability

The original contributions presented in the study are included in the article/supplementary material, further inquiries can be directed to the corresponding authors.
